# Impact of Nanocapsules on Red Blood Cells Interplay Jointly Assessed by Optical Tweezers and Microscopy

**DOI:** 10.3390/mi11010019

**Published:** 2019-12-23

**Authors:** Tatiana Avsievich, Yana Tarakanchikova, Ruixue Zhu, Alexey Popov, Alexander Bykov, Ilya Skovorodkin, Seppo Vainio, Igor Meglinski

**Affiliations:** 1 Optoelectronics and Measurement Techniques Research Unit, University of Oulu, 90014 Oulu, Finland; yana.tarakanchikova@oulu.fi (Y.T.); ruixue.zhu@oulu.fi (R.Z.); alexander.bykov@oulu.fi (A.B.); 2 Nanobiotechnology Laboratory, St. Petersburg Academic University, St. Petersburg 194021, Russia; 3 RASA Center in St. Petersburg, Peter the Great St. Petersburg Polytechnic University, St. Petersburg 195251, Russia; 4 Biocenter Oulu and Faculty of Biochemistry and Molecular Medicine, Biocenter Oulu, Laboratory of Developmental Biology, University of Oulu, 90014 Oulu, Finland; ilya.skovorodkin@oulu.fi (I.S.); seppo.vainio@oulu.fi (S.V.); 5 InfoTech Oulu, Borealis Biobank of Northern Finland, Oulu University Hospital, University of Oulu, 90014 Oulu, Finland; 6 Interdisciplinary Laboratory of Biophotonics, National Research Tomsk State University, Tomsk 634050, Russia; 7 Institute of Engineering Physics for Biomedicine (PhysBio), National Research Nuclear University MEPhI, Moscow 115409, Russia; 8 Aston Institute of Materials Research, School of Engineering and Applied Science, Aston University, Birmingham B4 7ET, UK; 9 School of Life & Health Sciences, Aston University, Birmingham B4 7ET, UK

**Keywords:** polymeric nanocapsules, red blood cells, optical tweezers, SEM, optical microscopy, cytotoxicity, human mesenchymal stem cells

## Abstract

In the framework of novel medical paradigm the red blood cells (RBCs) have a great potential to be used as drug delivery carriers. This approach requires an ultimate understanding of the peculiarities of mutual interaction of RBC influenced by nano-materials composed the drugs. Optical tweezers (OT) is widely used to explore mechanisms of cells’ interaction with the ability to trap non-invasively, manipulate and displace living cells with a notably high accuracy. In the current study, the mutual interaction of RBC with polymeric nano-capsules (NCs) is investigated utilizing a two-channel OT system. The obtained results suggest that, in the presence of NCs, the RBC aggregation in plasma satisfies the ‘cross-bridges’ model. Complementarily, the allocation of NCs on the RBC membrane was observed by scanning electron microscopy (SEM), while for assessment of NCs-induced morphological changes the tests with the human mesenchymal stem cells (hMSC) was performed. The combined application of OT and advanced microscopy approaches brings new insights into the conception of direct observation of cells interaction influenced by NCs for the estimation of possible cytotoxic effects.

## 1. Introduction

Nowadays, there are numerous delivery systems such as: natural biological vehicles (such as bacteria and viruses [1,2], various cell types including red blood cells (RBCs) [3,4,5], immune cells [6,7], stem cells [8,9,10,11] and manufactured carriers (liposomes [12], micelle [13], polymeric capsules [14,15,16,17], polymeric complexes [18,19,20,21]). Polymeric nanocapsules (NCs) are a promising system of natural carriers for targeted drug delivery because of their low cytotoxicity, shell flexibility, and high in vivo stability [22,23,24,25,26]. A wide range of drugs [27,28,29] and nucleotides of ribonucleic acid (RNA)/deoxyribonucleic acid (DNA) [30,31,32] can be effectively encapsulated in the polymer capsules. Effective distribution of drugs in target tissues can be achieved by active delivery methods that are potentially much more beneficial than passive accumulation. This is why design of new drug carriers is one of the fastest developing directions of research in the nanomedicine area. This allows, in particular, creating a controllable photosensitizer delivery system for cell applications [33,34,35]. Increasing use of nanomaterials, including nanoparticles and NCs in household, medical and industrial products requires a careful assessment of possible consequences [36,37,38].

A promising area of targeted delivery of physiologically active substances implies the use of polymeric NCs for transport of medical drugs through the blood vessels. However, there is a potential risk of negative impact of NCs on blood cells [39]. RBCs, being the most abundant blood component, easily obtainable and available, remain one of the most common test systems for characterizing the action of biologically active compounds at the cellular and subcellular levels [40]. For administration of NCs into a human organism the bloodstream serves as a common pathway for delivering medicine (including nanodrugs), but it remains largely unknown and hardly predictable, whether nanoparticles affect blood components. Intravenous injection assumes circulation of NCs in a blood stream for a certain time. During this period, the injected drugs should be delivered to targeted tissues with minimized adverse influence or toxic effects on organs and systems used as a delivery pathway.

The understanding of NCs’ behavior in close to natural conditions will help to predict the possible complications and undesirable side effects of NC-based drug delivery systems. Another promising direction is the development of hybrid delivery systems, when a cell is coupled with an active component [41,42], e.g., NCs, thus increasing the effectiveness of the delivery and ensuring prolonged circulation of the drug in the organism. Therefore, revealing the influence of NCs on cells a single-cell level, can bring new insights, thus helping to improve biocompatibility of NCs.

RBC aggregation is a reversible process of adhering RBCs to each other in a face-to-face morphology, which depends on the blood plasma composition (macromolecules, including plasma proteins) and RBC cellular properties [43]. Aggregation of RBCs affects blood viscosity and plays a crucial role in the blood microcirculation [44]. Its characteristics are sensitive to the majority of socially significant diseases, such as diabetes, inflammation, cardiovascular embolism, myocardial ischemia etc. [45], hence RBC aggregation state can be used as a marker of the organism physiological status. In the present research RBC aggregation is evaluated through the mutual RBCs interactions between the individual single cells.

The optical tweezers (OT) technique allows for manipulation of microscopic objects including cells with a strongly focused laser beam and for measuring interaction forces between them within 0.1–100 pN. The method was first introduced by A. Ashkin [46,47], who was awarded the Nobel Prize in Physics for this development in 2018. OT method has been applied for a variety of applications in the field of physics and biology. The method was efficiently used in RBCs studies for measuring aggregation forces between individual interacting cells and to demonstrate possibility of performing experiments in the natural state of RBCs without impairing their properties. A recent study demonstrates successful measurements of such forces in autologous plasma and in protein solutions [48].

The present study focuses on the evaluation of RBC response to NCs in terms of RBC aggregation in autologous plasma using OT, complemented with SEM of RBCs. Additionally, microscopic observations of changes in morphology of human mesenchymal stem cells (hMSCs) were performed. HMSCs represent a significantly different type of cells compared to RBCs, being less deformable, phagocytic (able to engulf other cells or particles) and having a nucleus. A recent study [42] demonstrated that hMSCs cells coupled with NCs can serve as an effective antitumor drug delivery system, underlying the importance of understanding the NC-cell interaction.

## 2. Materials and Methods

### 2.1. Experimental Setup (Optical Tweezers)

**Figure 1:**
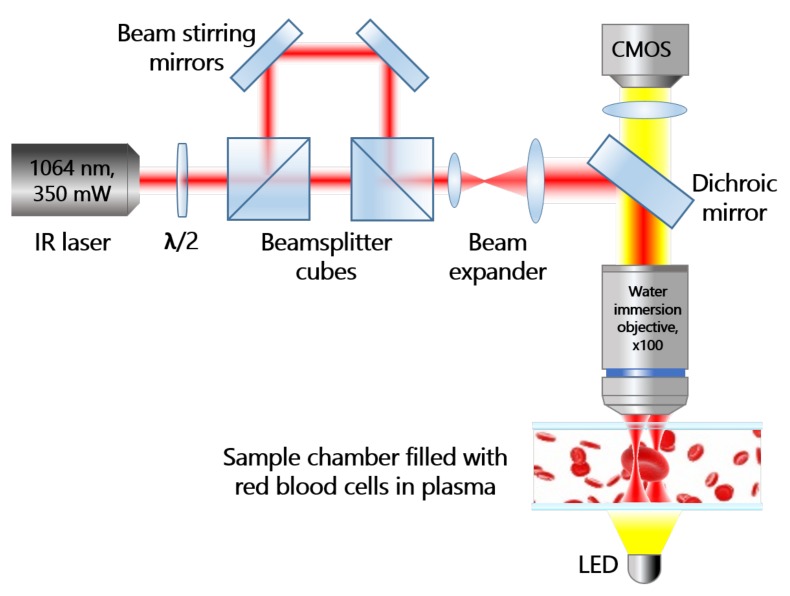
Schematic representation of the Optical tweezers (OT) setup. Optical traps are formed inside the sample chamber with a water immersion objective with high numerical aperture (100×,NA=1). Measurement procedure was imaged in the transmission mode and recorded with the complementary metal-oxide-semiconductor (CMOS) camera.

The OT system (Figure 1) forms two optical traps and allows for simultaneous manipulation of two RBCs. The traps are formed by a Nd:YAG infrared laser ILML3IF-300 (Leadlight Technology, Hsin-Chu, Taiwan) beam with output power up to 350 mW at a wavelength of 1064 nm.

The overall power of the initial beam is controlled by a half-wave plate λ/2 and a first beamsplitter cube; the power of the individual beams is regulated by a combination of λ/2 plates (not shown) and the second beamsplitter cube. The position of the second trap inside the sample in the focal plane of the focusing objective is adjusted by a conjugated beam tuneable mirror. After passing through the beam expander, light reaches the dichroic mirror, which reflects the laser beam to the sample. Tightly focusing of the laser beam was realized by an objective lens of high numerical aperture (NA = 1.0, water immersion LUMPlanFl, lOlympus, Tokyo, Japan), forming two traps inside the sample chamber. The sample illuminated with a white LED is transmitted through the infrared filter (IR-filter) to the complementary metal-oxide-semiconductor (CMOS) camera (Pixelink PL-B621M, lPixelink, Ottawa, Canada).

To calibrate the OT a viscous friction method was applied based on Stokes’ law. The drag force is

(1) $$F_{d}=6\pi \eta RvK,$$

where η is the dynamic viscosity of blood plasma (1.2 × 10 mPa·s) [49], r is the RBC radius considering an RBC to be an equivolume sphere with r = 2.7 μm, ν is the flow velocity relative to the object (velocity of the XY-stage movement), *K* is the correction factor for the ellipsoid.

Experiments were conducted at a 30-μm distance above the bottom of the cuvette to avoid interaction with other RBCs and bottom friction. The aggregation force was matched with the trapping force. The power at the moment when an RBC escapes the trap at a given velocity of the flow is equal to the trapping force.

### 2.2. Measurement Procedure

Measurements were performed as in our previous studies of mutual interaction of RBCs [50]. The aggregation force Fa of RBC is the minimum force required to stop two RBCs from overlapping. For a single measurement, two RBCs were trapped with individual optical traps, lifted for 30 μm and brought in contact at a small overlapping distance of Δx ≈2μm. From this stable position, the power of one trap starts slowly decreasing until spontaneous aggregation takes place and RBCs overlap. The power of the attenuated laser beam registered at this moment is equal to the aggregation force between the two RBCs. Disaggregation force (the force applied to separate two overlapped RBCs) was also measured to estimate energy density of RBC interaction.

### 2.3. Preparation of RBC Samples

Blood from the same donor was drawn by venepuncture directly in (EDTA)-covered tubes to prevent coagulation. The experiments were conducted in accordance with the obtained ethical permission from the Finnish Red Cross (No. 11/2019). In order to obtain platelet-free plasma, the whole blood was centrifuged at 3000× *g* for 10 min, plasma supernatant was aspirated gently through a micropipette and the procedure was repeated to remove the rest of the RBCs. Experiments were conducted at room temperature (20 ${}^{\circ }$C).

RBCs were collected prior to experiments by finger pricking from a healthy donor (the same person was used as for plasma retrieval). A written consent was obtained from the volunteer. RBCs from the blood sample (hematocrit 45%) were washed with Dulbecco’s Phosphate Buffered Saline (PBS, pH 7.4) by centrifugation at 4000× *g* for 10 min. Sedimented RBCs were accurately taken from the vial and suspended in blood plasma at a concentration of 0.5%.

NCs were dispersed in distilled water and placed in an ultrasonic bath for 5 min to destroy aggregates. Then NCs were added to the RBCs suspended in plasma following incubation for 1 h. The concentration of NCs was high enough to ensure interaction of RBCs with NCs—0.050106 per μL (with RBC concentration of 0.045 × 10${}^{6}$ per μL). NCs were easily visible via the objective 100× during the experiments.

A sample chamber from a glass slide with a glass coverslip separated by a double-sided adhesive tape to form a 0.1-mm-thick sample gap was used in the measurements. Opened edges of the cuvette were isolated with Vaseline to prevent drying of the sample and the thermal drift. The plasma suspension of RBCs incubated with NCs was pipetted into the sample chamber to perform measurements.

### 2.4. Synthesis of Nanocapsules

The following chemicals obtained from Sigma-Aldrich were used to synthesize NCs: dextran sulfate, sodium salt (DS, MW > 70,000), poly-L-arginine hydrochloride (PARG, MW > 70,000), phosphate buffered saline (PBS, 0.01 M), calcium chloride dihydrate, ethylenediaminetetraacetic acid (EDTA) disodium salt, rhodamine ltetramethylrhodamine (TRITC) isothiocyanate (MW 536.08), anhydrous sodium carbonate, sodium chloride, citrate-stabilized magnetite nanoparticles (FeNPs), goat anti-Mouse IgG2a Secondary Antibody labeled by Alexa Fluor 546 (A-21133, Thermo Fisher Scientific, Waltham, MA, USA). Control siRNA labelled with Alexa 546 were purchased from Qiagen. Synthesis of ${\mathrm{Fe}}_{3}O_{4}$ nanoparticles was performed using a home-built setup similar to that described by German et al. [15].

**Figure 2:**
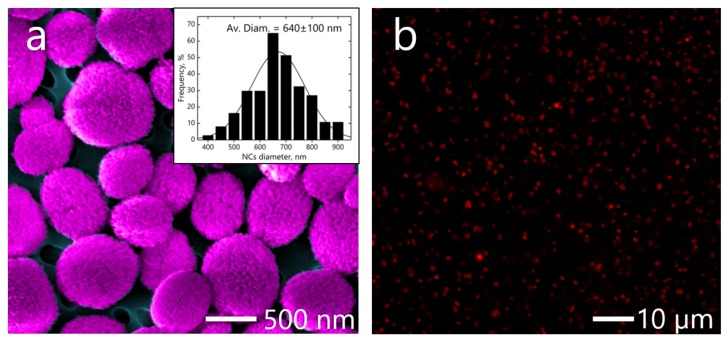
(**a**) Coloured scanning electron microscopy (SEM) images of core-shell polymeric nano-capsules (NCs) with size distribution with Gaussian fitting. (**b**) The red fluorescence on the confocal image comes from the shell layers containing rhodamine-ltetramethylrhodamine (TRITC).

NCs were prepared using calcium carbonate (${\mathrm{CaCO}}_{3}$) particles as a sacrificial template. The ${\mathrm{CaCO}}_{3}$ particles were fabricated as before [16]. ${\mathrm{CaCl}}_{2}$ and ${\mathrm{Na}}_{2}{\mathrm{CO}}_{3}$ aqueous solutions (0.33 M) were mixed under vigorous stirring for 3 h and were dissolved in 10 mL ethylene glycol, leading to precipitation of ${\mathrm{CaCO}}_{3}$ particles. When finished, ${\mathrm{CaCO}}_{3}$ particles were washed with deionized water to remove unreacted species. The spherical ${\mathrm{CaCO}}_{3}$ submicron particles with an average diameter of 400–600 nm were prepared. NCs were manufactured using the layer-by-layer (LbL) assembly technique by alternative deposition of oppositely charged polyelectrolytes on the ${\mathrm{CaCO}}_{3}$ particles. For the layers, biocompatible polyelectrolytes such as dextran sulfate (DS) with the concentration of 1 mg/mL (2 mL) and poly-l-arginine hydrochloride (PARG) of 1 mg/mL (1 mL) were used. The average size of the core-shell capsules calculated from scanning electron microscopy (SEM) images was 640±100 nm, shown on Figure 2a). The capsules were labelled by Rhodamine TRITC dye isothiocyanate and RNA molecules were adsorbed on the $CaCO_{3}$ surface. Encapsulation of the dye was performed during LbL. Fluorescence of the loaded Rhodamine TRITC dye was measured by confocal microscopy (see Figure 2b).

Four types of polymeric core-shell NCs produced from the $CaCO_{3}$ particles, differed by the material embedded into the shells, were tested in the present study:

1. Secondary antibody NCs are NCs labeled with secondary antibody anti-mouse Alexa Fluor 546. NCs are often covered with antibodies to provide immuno-specific binding to the target cells [51].
2. RNA-NCs are NCs with RNA-labeled Rhodamine-TRITC, widely used for cancer theranostics, and can be applied for genome editing [52,53].
3. ${\mathrm{Fe}}_{3}O_{4}$ magnetite NCs are NCs loaded with magnetic particles, which allow the magnetically assisted delivery, controlled drug release, and MRI imaging [54].
4. Rhodamine-labelled NCs are NCs labeled only with Rhodamine TRITC in a shell, which is used to control the allocation of NCs [16].

### 2.5. Preparation of RCBs for Optical and Scanning Electron Microscopy (SEM)

Washed RBCs were incubated with NCs in PBS at the concentration mentioned above within 1 h followed by their fixation with 1% glutaraldehyde (lMerck, Kenilworth, NJ, USA) for 15 min. The fixative solution was then removed from the vial and replaced with distilled water. A droplet of RBC suspension was pipetted onto a paper filter attached to a carbon tape. After evacuation the dried samples were coated with a 5-nm-thick platinum layer for electron microscopy (Zeiss Ultra Plus and Sigma FESEM, lCarl Zeiss, Oberkochen, Germany).

### 2.6. Estimation of the Human Mesenchymal Stem Cells (HMSCS) Morphological Properties

Human mesenchymal stem cells (hMSCs) were obtained from the bone marrow of healthy donors who gave their informed consent. Plating procedure was applied for cell isolation: 1 mL of heparinized bone marrow was resuspended in an α minimum essential medium (Lonza, Basel, Switzerland) supplemented with 100 IU/mL penicillin, 0.1 mg/mL streptomycin (lBiolot, Saint Petersburg, Russia), 10 vol % fetal bovine serum (FBS, HyClone, Logan, UT, USA), and 2 mM UltraGlutamine I (Lonza, Switzerland). The cells were cultured in Dulbecco’s Modified Eagle Medium (DMEM) supplemented with 10% inactivated Fetal Bovine Serum (lThermo-Fischer Scientific, Waltham, MA, USA), under standard conditions (37 ${}^{\circ }$C, 5% of ${\mathrm{CO}}_{2}$, humidified sterile environment) to >85% confluency. For estimation of the cell morphological properties, the cells were incubated with NCs for 24 h; then they were fixed with 4% paraformaldehyde solution for immunostaining.

## 3. Results

Synthesized NCs differed only by the material loaded into the capsule’s shell. Loaded material have no effect on a surface charge of NCs, since measured zeta potential value is 40 mV for all the NCs types. This value is associated with good or moderate stability of NCs in water solutions. The particular interest of the study is if the human blood plasma affecting NCs properties. OT measurements of RBC were performed in vitro in autologous blood plasma mimicking in vivo conditions.

**Figure 3:**
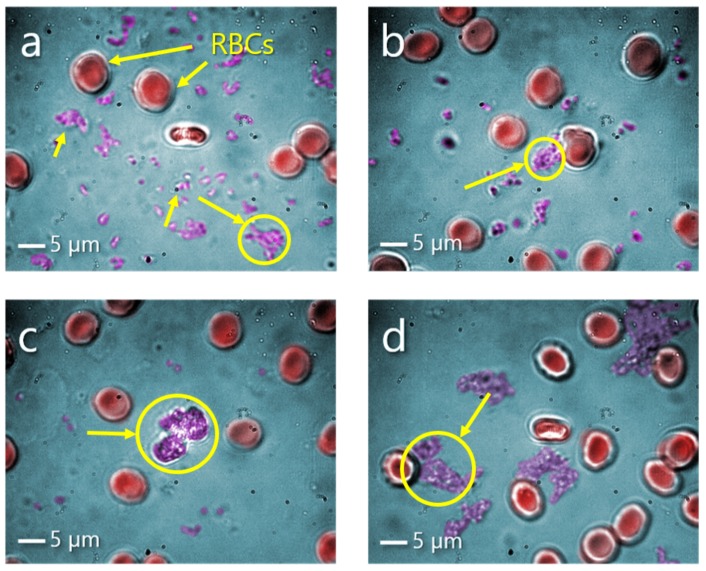
Optical microscopy images (objective 100×) of lred blood cells (RBCs) in blood plasma with NCs in false color (NC aggregates are encircled) during measurements with OT: (**a**) Secondary antibody NCs; (**b**) lribonucleic acid (RNA) NCs; (**c**) ${\mathrm{Fe}}_{3}O_{4}$ NCs; (**d**) rhodamine-labelled NCs.

After incubation of red blood cells (RBCs) with NCs in buffer solution, sample was diluted in blood plasma. NCs being transferred into blood plasma tend to form agglomerates, which are visible and easily distinguished in the sample (Figure 3a–d). Magnetite ${\mathrm{Fe}}_{3}O_{4}$ and rhodamine-labelled NCs have formed the biggest aggregates among observed, exceeding the size of RBCs and reaching 10–15 μm, encircled on the Figure 3c,d. While NCs loaded with secondary antibody and RNA formed smaller aggregates and exhibited an adequate stability in blood plasma.

**Figure 4:**
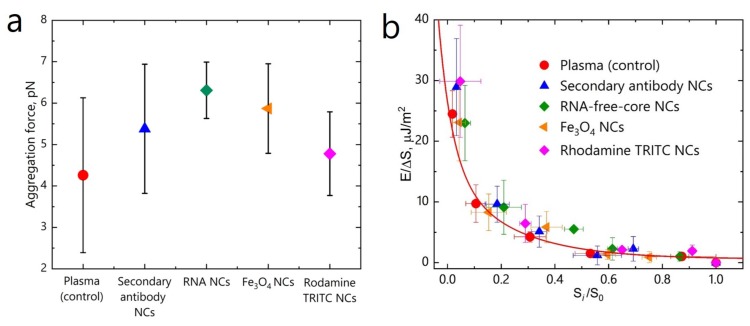
RBC aggregation force (**a**) and interaction energy density dependence on interaction area (**b**) between RBCs in blood plasma alone (control) and in presence of NCs measured with OT. Δ S is conjugated interaction area, $S_{i}/S_{0}$ is relative displacement of RBCs from the initial overlapping area $S_{0}$. The red curve corresponds to the cross-bridges model.

Aggregation forces between RBCs were measured on 20 pairs of cells for each sample. The measurements of the aggregation forces between RBCs showed that compared to the control measurements in the blood plasma without NCs treatment ($F_{a}$ = 4.26±1.87 pN), no statistically significant differences (Mann-Whitney U-test at *p* < 0.05) were found after RBCs incubation with NCs, regardless the type of NCs. The aggregation force between RBCs was found to be almost the same for all samples (Figure 4a), $F_{a}$ = 5.38±1.56 pN for the secondary antibody NCs, $F_{a}$ = 6.31±0.68 pN for RNA NCs, $F_{a}$ = 5.87±1.08 pN for ${\mathrm{Fe}}_{3}O_{4}$ NCs, and $F_{a}$ = 4.78±1.01 pN for Rhodamine-labelled NCs.

Interaction energy density between RBCs was calculated as described before [48]. The obtained dependence demonstrates the growth of the interaction energy between RBCs upon their separation with OT (Figure 4b). This dependence is typical for the so called “cross-bridges” model, assuming the accumulation of “cross-bridges” formed by plasma proteins between the interacting RBCs. RBCs interaction energy always increases upon cell disaggregation, as it was confirmed before in our studies [50,55,56]. Here, RBCs in presence of all the types of NCs demonstrate the same dependency, suggesting no difference in the interaction mechanism between the cells.

**Figure 5:**
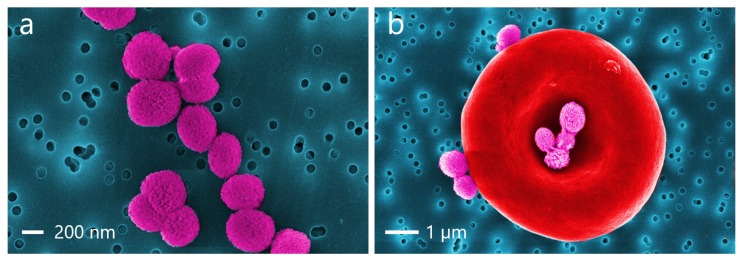
Coloured SEM images of (**a**) core-shell NCs with RNA; (**b**) rhodamine-labelled NCs attached to a RBC.

Analyzing SEM images of RBCs incubated with NCs (approximately 100s of cells), no visible adverse effects on the RBC morphology were found. NCs are too big to be able to penetrate RBCs and the only expected way of NCs influence on RBC is through the interaction/attachment of NCs to the cell membrane, possibly causing membrane deformations or ruptures. According to the SEM images RBCs preserved their native shape even with few NCs being found attached to a single cell (Figure 5b). Besides, no visible changes of the RBC shape were observed during OT measurements with 100× magnification.

**Figure 6:**
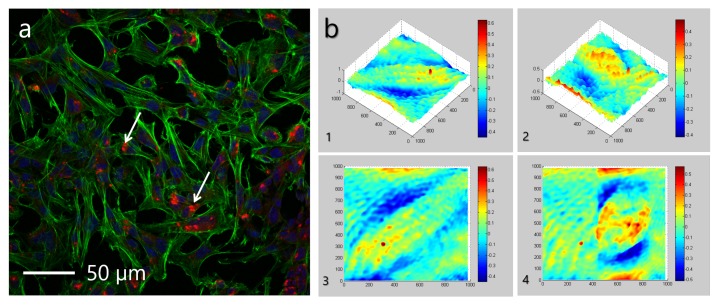
Interaction of polymeric NCs with Human Mesenchymal Stem Cells (hMSCs) elucidated with optical confocal microscopy. (**a**) Confocal image of hMSCa after 24 h incubation with NCs, where the green color denotes phalloidin lfluorescein (FITC) and the blue color denotes 4’,6-diamidino-2-phenylindole (DAPI) dyes. The arrows indicate fluorescing rhodamine-labelled NCs; (**b**) Phase images of hMSCs without capsules - initially: 3D (1), 2D (3) and after 24 h incubation with capsules: 3D (2), 2D (4).

When NCs are incubated with hMSC, as seen in Figure 6a, changes in the cell structure were not observed. hMSCs are significantly larger cells of 20–30 μm, comparing to RBCs, and NCs can potentially penetrate the cell. Phase images (see Figure 6b) allow for evaluating the cell structure integrity. Capsules possess neutral pH of about 7, which is equal to the cell’s pH, implying the biocompatibility of NCs. This observation proves the idea that NCs do not possess the adverse cytotoxic effect on the morphological properties of cells.

## 4. Discussion

Since NCs carry positive charge, the preferable interaction with the negatively charged RBC membranes was expected. However, in blood plasma due to the presence of different proteins contributing to complex interaction of NCs with the surrounding media, NCs surface properties change. This results in the strong agglomeration observed for magnetite ${\mathrm{Fe}}_{3}O_{4}$ and rhodamine-labelled NCs.

The tendency of NCs for agglomeration significantly decreases interaction between RBC membranes and NCs. In this way aggregation forces between the cells will be the same as in the control measurements. Interaction mechanism between RBCs have not changed for the cells incubated with NCs. Plasma protein adsorption can lead to the change of the NC surfaces properties, forming so-called “corona”, which is able to decrease cytotoxic effects of the positively charged nanoparticles [57]. Thus, even if ${\mathrm{Fe}}_{3}O_{4}$ and Rhodamine-labelled NCs do not cause RBC aggregation, strong NCs self-aggregation can reduce the efficiency of NCs injected into the blood stream and potentially cause blockage of small vessels. After incubation of negatively charged hMSCs with NCs, the latter were internalized inside the cell’s matrices through the hMSC phagocytosis mechanism. Based on above, NCs charge can be considered as the defining mechanism for NC-cell interactions, due to the implications introduced by media (plasma) or cell intrinsic properties.

Incubation of RBCs with NCs in PBS resulted in the attachment of NCs to an RBC membrane, forming RBC-NCs complex, as it follows from the SEM. Probably, interaction between the cell and NCs is weak, so the complexes are falling apart and being transferred into blood plasma. For the desired coupling of RBCs with NCs proper NCs functionalization should be addressed. Regardless the material being embedded into the NCs shell, NCs show no influence of RBC interaction properties.

NCs must meet basic requirements for medical applications such as biocompatibility and ability to reach a target tissue. The described OT technology allows for fast evaluation of NC influence on the aggregation state of individual RBCs. The obtained information enables finding safe concentrations of NCs to prevent their adverse influence on blood rheological properties, to reveal the influence o material loaded into the NCs on cell state, decreasing in this way the risk of side effects.

For the integral assessment of NCs, other blood components should be also involved, such as platelets and leukocytes. OT and microscopy based approaches conjugated with microfluidic platforms, can be transformed into the blood vessel modelling system, to reveal the effects of different nanomaterials on cells, minimizing the use of animals for testing.

The use of fluorescence spectroscopy, in combination with phase microscopy, can also increase the statistical significance and reliability of clinical trials and reduce the number of animals needed and decrease their suffering. The data obtained by this method provides information about the pharmacodynamics and the optimal dosage of the drug. To carry out clinical trials, it is necessary to estimate the capsules influence on the morphology of cells. HMSCs after long incubation with NCs preserved their integrity and viability, despite the evenly distributed NCs inside the cells.

## 5. Conclusions

OT and optical microscopy observations have demonstrated different behavior of NCs in blood plasma. The magnetite and rhodamine-labelled NCs are prone to strong agglomeration in plasma, while secondary antibody and RNA-NCs are relatively stable and uniformly distributed. NCs self-aggregation can potentially result in a microvascular blockage of small blood capillaries in vivo. None of the tested NCs caused changes of aggregation forces of RBCs compared to the control measurements. In all cases, the cross-bridges model of RBC interaction is applicable, confirming the same interaction mechanism between RBCs in presence of NCs. Combination of OT and SEM imaging together with conventional optical microscopy shows that none of the types of NCs used have any adverse influence on RBCs’ morphology, or on hMSCs, indicating that polymeric NCs are not cytotoxic for these cells in the tested conditions.

## Abbreviations

The following abbreviations are used in this manuscript:

OT	Optical tweezers
NCs	Nanocapsules
RBCs	Red blood cells
hMSCs	human mesenchymal stem cells
